# Mindfulness-Based Cognitive Therapy for Trichotillomania: A Bayesian Case-Control Study

**DOI:** 10.5334/pb.bj

**Published:** 2015-07-10

**Authors:** Alexandre Heeren, Charlotte Busana, Charlotte Coussement, Pierre Philippot

**Affiliations:** 1 Laboratory for Experimental Psychopathology, Psychological Sciences Research Institute, Université catholique de Louvain 10, Place du Cardinal Mercier – 1348 Louvain-La-Neuve, Belgium

**Keywords:** Trichotillomania, Mindfulness-based interventions, Hair-pulling, Single-case, Bayesian approach

## Abstract

Over the last years, mindfulness-based interventions combined with habit reversal training have been demonstrated to be particularly suitable for addressing trichotillomania. However, because these studies always combined mindfulness training to habit reversal without including either a mindfulness or habit reversal condition alone, it is still unclear whether clinical benefits are the consequences of mindfulness or merely result from habit reversal training. The primary purpose of the present study was thus to examine whether a mindfulness training procedure without habit reversal could alleviate trichotillomania. Using a Bayesian probabilistic approach for single-case design, client’s hair loss severity and level of mindfulness were compared to a normative sample (*n* = 15) before treatment, after treatment, and at six-month follow-up. Improvement in mindfulness first occurred, and that beneficial effect then transferred to hair-pulling. Indeed, as compared to the normative sample, the client exhibited, from baseline to post-treatment, an improvement in mindfulness. Although a marginal trend to improvement was already evidenced at post-treatment, the mindfulness program only had a significant beneficial effect transferred to hair-loss severity at six-month follow-up. Although it remains particularly difficult to infer generalization from one client, the data from the present case study are the first to suggest that mindfulness training per se might be a suitable clinical intervention for trichotillomania.

## Mindfulness-based Cognitive Therapy for Trichotillomania: A Bayesian Case-control study

Trichotillomania (TTM) or hair-pulling disorder is a psychological disorder characterized by the recurrent pulling of one’s own hair resulting in notable hair loss (Amercian Psychiatric Association, 2013). During hair-pulling the avulsion of hair occurs mostly from the scalp, but commonly from eyebrows, eyelashes, beard, and pubic areas (Duke, Keeley, Geffken, & Storch, 2010; Woods et al., 2006a). In addition to these criteria, the person with TTM must experience tension prior to pulling that is relieved after the pulling episode, or must experience an increasing sense of tension during attempts to refrain from pulling. Lifetime prevalence has been estimated to range from 0.6% to 1% (e.g., Duke et al., 2010). TTM results in significant impairments across a variety of domains including occupational functioning and clinically significant levels of anxiety, depression, and stress (Flessner et al., 2008; [Bibr B26][Bibr B67]). TTM also often leads to potentially several medical complications, such as skin irritation, scalp irritation, or trichobezoars (e.g., Duke et al., 2010; Keuthen, Stein, & Christensen, 2001).

Neurocognitive studies of adults with TTM have shown deficits in inhibitory control (Bohne, Keuthen, Tuschen-Caffier, & Wihlemen, 2005a; Bohne, Savage, Deckersbach, Keuthen, & Wilhem, 2008; Chamberlain, Blackwell, Fineberg, Robbins, & Sahakian, 2006; Lee, Franklin, Turkel, Goetz, & Woods, 2012; Stanley, Hannay, & Breckenridge, 1997). Inhibitory control is best characterized as the ability to inhibit prepotent courses actions and resistance to interference from irrelevant stimuli (Bjorklund & Harnishfegar, 1995; Nigg, 2000). Neuroimaging studies have reported that TTM patients have impaired activations of the fronto-striatal pathway (i.e., putamen, caudate, cingulate anterior cortex, prefrontal cortex) during tasks involving such an inhibitory control (Chamberlain et al., 2008, 2010; O’Sullivan et al., 1997; Stein et al., 2002; Swedo et al., 1991).

The traditional behavioral approaches to TTM include habit reversal training (HRT) i.e. training a competing response (e.g., fist clenching) that is incompatible with and blocks the hair-pulling response. In a seminal study, Azrin, Nunn, and Frantz (1980) randomly allocated 34 TTM patients to a two-hour treatment session including either HRT or negative practice (i.e., behavioral technique in which the patients imitate pulling movement in front of mirror without actually pulling). They found that those patients in the HRT condition evidenced significant reductions in hair-pulling episodes and higher remissions rates. At four-week follow-up, 74% of the TTM patients in the HRT condition no longer reported hair-pulling, as compared to 33% in the negative practice condition. Since this initial publication, several studies have reported that habit reversal is an empirically validated and well-accepted treatment for TTM patients (for a recent meta-analysis, see Bloch et al., 2007).

However, over the last decades, several studies have suggested that TTM patients may also benefit from psychotherapeutic approaches that directly target emotion regulation as TTM patients often performed hair-pulling to down regulate negative inner experience (Diefenbach, Tollin, Meunier, & Worhunsky, 2008; Grant, Odlaug, & Potenza, 2007; Shusterman, Feld, Baer, & Keuthen, 2009; Teng, Woods, Twohig, & Marcks, 2002). As a consequence, practitioners have progressively started to add components of usual cognitive and behavioral approaches to TTM treatment that aimed at modifying contextual stimuli as well as cognitions that both trigger hair-pulling (e.g., Mansueto, Townsley-Stemberger, McCombs-Thomas, & Goldfinger-Colomb, 1997; Ninan, Rothbaum, Marsteller, Knight, & Eccard, 2000; Pelissier & O’Connor, 2004).

Over the last years, mindfulness-based interventions (MBI) combined with HRT have been demonstrated to be particularly suitable for addressing TTM (Crosby, Dehlin, Mitchell, & Twohig, 2012; Keuthen et al., 2010, 2011; Twohig & Woods, 2004). Mindfulness is defined as the ability to bring one’s attention to experience in the present moment in a nonjudgmental way (Kabat-Zinn, 1990). In recent years, several meta-analyses examining the efficacy of MBI have concluded that these interventions may help to alleviate a variety of mental health problems, including anxiety, depression, and stress disorders, and improve overall psychological functioning (Baer, 2003; Grossman, Niemann, Schmidt, & Walach, 2004; Khoury et al., 2013).

To date, two kinds of MBI have been used in conjunction with the traditional HRT to treat TTM: *Acceptance and Commitment Therapy* (ACT; Hayes, Strosahl, & Wilson, 1999) and *Dialectical Behavior Therapy* (DBT; Linehan, 1993). Both of these approaches specifically address the role of acceptance and mindfulness processes to decrease maladaptive emotion regulation and produce behavior change with respect to client values.

Regarding the combination of ACT and HRT, several studies have reported preliminary evidence. Twohig and Woods (2004) found that a seven-session program combining ACT and HRT was successful in decreasing hairs pulled to near-zero levels for four of six TTM patients, with three of them exhibiting gains maintenance at three-month follow-up. These findings were corroborated in a controlled randomized trial among 25 TTM patients comparing a 10-session treatment combining ACT and HRT to a waitlist condition (Woods, Wetterneck, & Flessner, 2006b). Sixty-six percent of participants in the treatment condition achieved clinically significant reductions in symptoms compared to 8% in the waitlist condition. A follow-up assessment indicated that these treatment gains were maintained at three-month follow-up.

Regarding the combination of DBT and HRT, Keuthen and collaborators (2010) reported an open clinical trial among 10 TTM patients. The treatment consisted of 11 weekly 50-minute acute treatment sessions followed by four boosting sessions during a maintenance phase over the subsequent three months (at 2, 4, 8, and 12 weeks post-treatment, respectively). Results showed that the treatment led to a significant improvement in hair-pulling severity, emotion regulation, anxiety, and depressive symptoms, with benefits maintained at three-month follow-up. More recently, in a follow-up study, Keuthen and colleagues (2011) also reported that these outcomes maintained at a six-month follow-up assessment and that improvement in hair-pulling correlated with changes in emotion regulation.

Taken together, these studies suggest that both ACT and DBT combined with HRT can help in reducing TTM. Nevertheless, uncertainty still abounds regarding whether these benefits can be unambiguously attributed to a change in mindfulness resulting from DBT and ACT as they both mixed mindfulness practice to some other therapeutic components (e.g., acceptance, emotion labeling). Moreover, as these studies always combined MBI to HRT without including either a MBI or HRT condition alone, it is still unclear whether these clinical benefits are the consequences of MBI or merely the results of HRT.

As a consequence, it remains particularly difficult to gauge the precise contribution of mindfulness *per se* in the improvement of TTM. This is unfortunate as it has been demonstrated that mindfulness training is particularly relevant to improve inhibitory control (Chiesa & Serretti, 2010; Heeren, Van Broeck, & Philippot, 2009; Tang et al., 2007), which has been argued to be a core neurocognitive mechanism involved in the maintenance of TTM (e.g., Bohne et al., 2005a; 2008; Chamberlain et al., 2006; Lee et al., 2012; Stanley et al., 1997). Moreover, it has been pointed out that TTM patients experience tension prior to pulling that is relieved after the pulling, leading, through operant conditioning, to the maintenance of a pulling behavior in response to the experience of such a tension (see above). As it has been showed that mindfulness training improves the ability to allow one’s feeling (pleasant and unpleasant) to come and go without reacting to them (Heeren et al., 2015), one might speculate that such a mindfulness practice per se might help to alleviate TTM.

The primary purpose of the present study was thus to examine whether a mindfulness training procedure without HRT could alleviate TTM symptoms within a single-case design. For clinical concerns, this issue is a critical one. Indeed, although the results from trials described above clearly suggest the efficacy of a combination of HRT and MBI for TTM, a critical issue vis-à-vis the application of mindfulness intervention in TTM is to establish its efficacy beyond its combination with other therapeutic components.

## Method

### Design and data analysis

An A-B design (Barlow & Hersen, 1984) with follow-up was implemented. During the baseline period, two therapists^1^ (AH and CB) met the client once in order to administer and collect the baseline measurements. Following the baseline, the same two therapists delivered the mindfulness training over an eight-week period. After the treatment, outcomes were assessed. Finally, the client returned to the clinical center six-month after the final training session for follow-up assessment.

The statistical recommendations for single-case design of Crawford, Garthwaite, and Porter (2010) were followed. A statistical Bayesian approach was used. There has been an explosion of interest in Bayesian statistical methods over the last decades (e.g., Heeren, Maurage, & Philippot, 2013). The main reason of this is the development of numerical techniques, notably Markov Chain Monte Carlo methods that have solved many of the remaining computational problems formally associated with the application of Bayesian analyses. Markov Chain Monte Carlo methods make inferences by generating a large number of observations from the distribution of the data (for a review, see Andrieu, De Freitas, Doucet, & Jordan, 2003). The essential difference between the classical and the Bayesian approaches is that the classical approach treats parameters as *fixed* but unknown, whereas, in the Bayesian approach, parameters are treated as *random* variables and hence have a probability distribution.

The single-case adaptation of Bayesian methods was used (Crawford & Garthwaite, 2007). First, this procedure provides a Bayesian hypothesis test. It estimates the probability that, according to a random selection, any individual of the 15 normative control participants would exhibit a larger difference than the single case, in either direction. Second, the Bayesian point estimate and 95% confidence interval for the abnormality of the case’s score are reported. The point estimate of the abnormality of the case’s score is the Bayesian estimated percentage of the normative sample that would obtain a score lower/higher than the case and the interval estimate quantifies the uncertainty over this percentage (Crawford, Garthwaite, & Howell, 2009). Data analyses were performed using SingleBayes_ES.exe (Crawford et al., 2010). This program implements Bayesian methods for comparison of a single case’s score to scores obtained in a normative sample.

### Participants

***Case report.*** DC is a right-handed 30-year-old Caucasian man, who lived with his girlfriend at the time of this study. He was a PhD student in humanities. He addressed himself to the Psychology Department Consulting Center with a complaint of hair-pulling. DC suffered from TTM since he was fourteen years old. His retrospective self-report of the beginning of his TTM did not evidence any traumatic or specific life events. Anamnesis revealed that he performed hair-pulling mainly with his right-hand over the ipsi- and contra-lateral eyebrows and the eyelashes section. Although DC reported that the intensity of hair-pulling increased with anxiety, stress and tiredness (physical and mental), he did not report any specific situation that may trigger hair-pulling. He explained the hair-pulling as a habit and did not feel any physical trigger. He had (a) no current substance abuse, (b) no current or past heart, respiratory, neurological problems, (c) no current or past use of psychotropic medications, (d) and was not currently engaged in any other form of psychological or psychiatric treatment. We obtained informed consent for publishing his data.

***Normative sample.*** In order to compare the performance of DC to a normative sample, a group was constituted by pairing DC to participants matched for age (+/– 12 months), gender, education level (post-graduate level) as well as laterality, but showing no TTM. We recruited 15 Belgian men among the acquaintances of the authors and the university employees. They were administrated the same measurements than DC. Their characteristics, as well as those of DC, appear in Table 1. In addition to the absence of TTM, all participants: (a) had no current substance abuse, (b) no current or past heart, respiratory, neurological problems, (c) no current or past use of psychotropic medications, (d) and were not currently engaged in any form of psychological and psychiatric treatment. Each participant was tested individually in a quiet room. We conducted the study in accordance with the ethical standards of the American Psychological Association. We obtained informed consent from each participant.

**Table 1:** Descriptive characteristics of the single-case to normative controls. *Note.* BDI = Beck Depression Inventory; STAI-Trait = State-Trait Anxiety Inventory – Trait version.

	Normative sample		Bayesian probability		Bayesian estimated percentage	
			
	*M*	*SD*	Case’s score	Probability (two-tailed)	Point	95% CI

Age	28.57	1.68	29	.81	59.61	39.55 − 77.85
BDI	6.00	7.73	12	.47	76.77	57.44 − 91.04
STAI-Trait	37.14	13.03	57	.17	91.87	77.51 − 98.82

### Assessment

***Self-report assessment.*** The Five Facet Mindfulness Questionnaire (FFMQ; Baer et al., 2008) was administered before and after treatment in order to assess change in mindfulness from baseline to post-treatment. The State-Trait Anxiety Inventory – Trait version (STAI-Trait; Spielberger, Gorsuch, Lushene, Vagg, & Jacobs, 1983), and the Beck Depression Inventory (BDI-II; Beck, Steer, & Brown, 1996) were administered before treatment in order to control for potential depressive symptoms as well as trait-anxiety.

*Five Facet Mindfulness Questionnaire* (*FFMQ*). Mindfulness was assessed using the FFMQ (Baer et al., 2008), a 39-item self-report measure assessing the level of mindfulness in daily life. It assesses five elements of mindfulness. These include *Observing* (i.e., attending to or noticing internal and external stimuli, such as emotions, cognitions, sights, sounds, or smells), *Describing* (i.e., noting or mentally labeling these stimuli with words), *Acting with awareness* (i.e., attending to one’s current actions, as opposed to behaving automatically or absent-mindedly), *Non-judgment of inner experience* (i.e., refraining from evaluation of one’s sensations, cognitions, and emotions) and *Nonreactivity to inner experience* (i.e., allowing one’s thoughts and feelings to come and go, without reacting to them). A Well-validated French version of the scale was used in the present study (Heeren, Douilliez, Peschard, Debrauwere, Philippot, 2011a).

*State-Trait Anxiety Inventory – Trait version* (STAI-Trait). STAI-Trait (Spielberger et al., 1983) is a 20-item self-report questionnaire assessing anxiety trait vulnerability). Well-validated French version of the scale was used in the present study (Bruchon-Schweitzer & Paulhan, 1993).

*Beck Depression Inventory* (BDI-II). The BDI (Beck et al., 1996) is a 21-item self-report measure of the symptoms of depression. Well-validated French version of the scale was used in the present study (Beck et al., 1998).

***Semi-structured interview.*** DC was also administrated the Mini International Neuropsychiatric Interview (Lecrubier, Weiller, Bonora, Amorin, & Lépine, 2002), a semi-structured interview assessing DSM-IV-TR axis I disorders. One therapist administrated the Mini International Neuropsychiatric Interview to DC. He had over three years of training in cognitive and behavioral therapy and one year of intensive training on using the Mini International Neuropsychiatric Interview to make reliable diagnoses. With the exception of TTM, results revealed that DC did not exhibit any DSM-IV axis 1 disorder. The second therapist (with at least three years of cognitive and behavioral therapy training) also attended to this semi-structured interview and corroborated these conclusions.

***Hair Loss Severity.*** In order to assess TTM symptoms severity, the hair loss severity was directly gauged through the observation of the bald spots. More specifically, we took pictures of DC’s hair-loss regions. For the normative sample, we took pictures of the same hair areas. Then, these pictures were submitted to two judges (i.e., a male and a female) who were blind to the goal of this study. They were asked to rate the hair areas of these pictures from 0 – *normal hair spots* (*normal hair area*) – to 100 – *large bald spots* (*abnormal hair area*). Inter-rater agreement was high (Spearman’ s rank *r* = 0.90, *p* < 0.001).

### Treatment

The mindfulness program consisted of eight weekly 90-minute individual treatment sessions. The initial treatment session was scheduled within two weeks after baseline. The mindfulness training was an adaptation of mindfulness-based cognitive therapy (Segal, Teasdale, & Williams, 2002). Mindfulness-Based Cognitive Therapy was designed for the prevention of depressive relapse. We adapted it for our case by modifying the psycho-education component of the program (sessions 4 and 5) to target stress, anxiety and depression rather than merely depression. Otherwise, all sessions and exercises followed the Mindfulness-Based Cognitive Therapy protocol [see Heeren & Philippot (2011b), for previous studies using such an adaptation of the program]. The program also included about 45-minute daily homework practice.

Two clinical psychologists delivered the training (AH and CB). One has a PhD in clinical psychology, a postgraduate training in cognitive and behavioral interventions, and had been trained in mindfulness-based psychological interventions. The other has a master degree in clinical psychology and was accomplishing a post-graduate clinical internship at the time of the study.

## Results

### Depression and anxiety at baseline

As shown in Table 1, DC showed a low-to-medium level of anxiety and a low level of depressive symptoms. However, these levels were not significantly different from the normative sample.

### Hair Loss Severity

**Figure 1:**
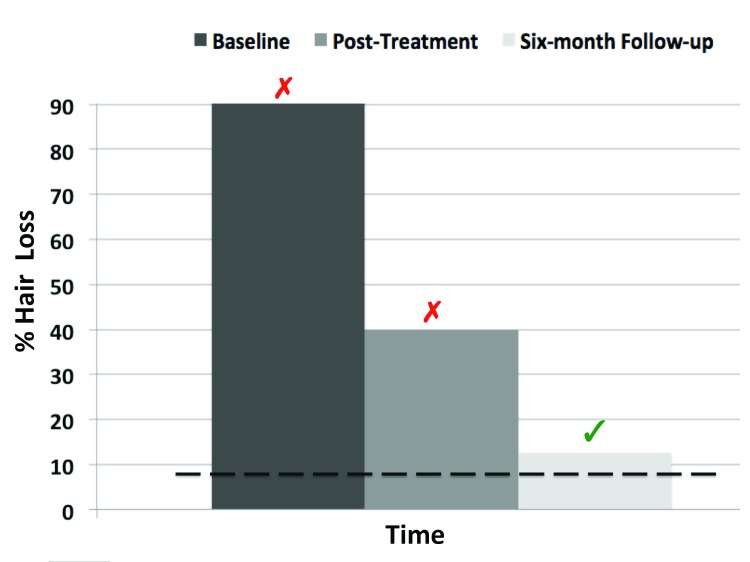
Changes in Hair Loss Severity as a function of Time. *Note:* The broken line depicts the mean score of the normative sample; the red “X” indicates that the case exhibits a deficit as compared to the normative sample; the green “v” indicates a significant restoration of this deficit.

At baseline, as shown in Table 2, it was estimated that 99.99% of the control population would obtain less bald spots than DC (95% CI = 99.90% to 100%). The score meets the criteria for a deficit: the null hypothesis that the score is an observation from the control population is rejected (*p* < .001). At post-treatment, it was estimated that 99.44 % of the control population obtain less bald spots than DC (95% CI = 99.31% to 100%). Again, the score meets the criteria for a deficit; that is, the null hypothesis is rejected (*p* = .011). However, at six-month follow-up, the null hypothesis, that the score is an observation from the normative sample, was not rejected (*p* = .67). DC’s change in hair-loss severity is shown in Figure 1.

**Table 2:** Case-controls score on hair loss. *Note:* A bold font emphasizes a significant difference between DC and the normative sample.

	Normative sample		Bayesian probability			Bayesian estimated percentage	
			
	*M*	*SD*	Time	Case’s score	Probability (two-tailed)	Point	95% CI

%Hair Loss	9.36	7.01	Baseline	92.5	< .001	99.99	99.90 – 100
			Post-treatment	40	.011	99.44	99.31 –100
			Six-month	12.5	.67	66.40	46.17 – 83.49

### Changes in mindfulness

**Figure 2:**
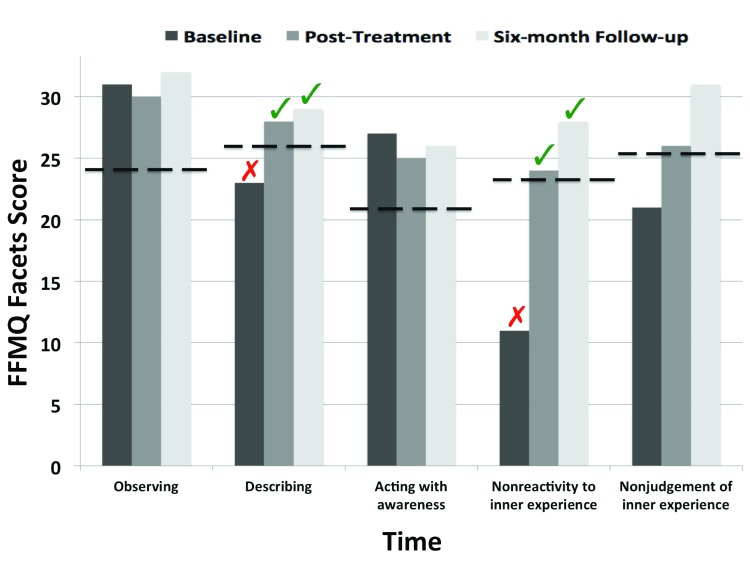
Changes in Mindfulness Facets as a function of Time. *Note:* The broken line depicts the mean score of the normative sample; the red “X” indicates that the case exhibits a deficit as compared to the normative sample; the green “v” indicates a significant restoration of this deficit.

At baseline, as shown in Table 3, the null hypothesis that the score is an observation from the normative sample was not rejected for the total FFMQ score (*p* > .05), suggesting that DC did not significantly differ from the normative sample. However, it was estimated that less than 5 % of the control population would differ from DC vis-à-vis *Describing* and the *Nonreactivity to inner experience* FFMQ facets. These scores meet the criteria for a deficit; that is, the null hypothesis that the score is an observation from the control population is rejected (*p* < .05). There were no such differences between DC and normative controls regarding other FFMQ facets. At post-treatment, however, the null hypothesis, that the score is an observation from the normative sample, was not rejected for both the total score and subscale scores (*p* > .05), suggesting the absence of difference between DC and the normative sample. At six-month follow-up, again, the null hypothesis that the score is an observation from the normative sample was not rejected for both the total score and subscale scores (*p* > .05), suggesting the absence of difference between DC and the normative sample. DC’s change in mindfulness are shown in Figure 2.

**Table 3:** Case-controls scores on mindfulness. *Note:* A bold font emphasizes a significant difference between DC and the normative sample.

Condition	Normative sample		Bayesian probability			Bayesian estimated percentage	
			
	*M*	*SD*	Time	Case’s score	Probability (two-tailed)	Point	95% CI

Observing	23.86	6.25	Baseline	31	.29	85.64	68.09 – 96.31
			Post-treatment	30	.36	82.17	63.79 – 4.40
			Six-month Follow-up	32	.23	88.58	72.30 – 97.61
Describing	26.00	1.59	Baseline	23	.04	4.40	0.31 – 15.44
			Post-treatment	28	.24	87.84	71.26 – 97.29
			Six-month Follow-up	29	.12	94.18	81.66 – 99.42
Acting with awareness	20.43	4.33	Baseline	27	.16	91.79	77.45 –98.79
			Post-treatment	25	.33	83.75	65.50 – 95.39
			Six-month Follow-up	26	.23	88.33	71.95 – 97.55
Nonreactivity to inner experience	23.64	4.89	Baseline	11	.02	1.26	0.01 – 6.59
			Post-treatment	24	.94	52.81	33.37 – 71.89
			Six-month Follow-up	28	.40	79.92	60.98 – 93.13
Nonjudgement of inner experience	25.14	7.14	Baseline	21	.58	29.18	13.14 – 49.10
			Post-treatment	26	.91	54.57	34.95 – 73.38
			Six-month Follow-up	31	.44	77.98	58.54 – 91.98
FFMQ score total	119.07	19.64	Baseline	113	.77	38.58	20.73 –58.69
			Post-treatment	133	.50	74.93	55.36 – 89.78
			Six-month Follow-up	146	.20	89.78	74.23 – 98.11

## Discussion

The primary purpose of this study was to examine whether a mindfulness training procedure without HRT could be successfully applied, in a regular clinical setting, to improve TTM. In accordance with our prediction, the results of this single-case study suggest that individuals who suffer from TTM may benefit from such a program, at least up to six months post-treatment.

First, as compared to the normative sample, DC exhibited, an improvement from baseline to post-treatment in two facets of mindfulness, namely Describing and Nonreactivity to inner experience. As mentioned above, previous research suggested that both inhibitory control and the inability to refrain hair-pulling in response to tension experienced prior to pulling are involved in the maintenance of TTM (e.g., Bohne et al., 2005b; 2008; Chamberlain et al., 2010; Lee et al., 2012; Stanley et al., 1997; Stein et al., 2002; Swedo et al., 1991). As the “Nonreactivity to inner experience” gauges the ability to allow one’s thoughts and feelings to come and go, without reacting to them, we may therefore infer that the present treatment targeted a core component involved in the maintenance of TTM. This finding is consistent with studies demonstrating that impoverished inhibitory control may play a critical role in the maintenance of TTM (Bohne et al., 2008; Chamberlain et al., 2006; Lee et al., 2012; Stanley et al., 1997). For instance, Bohne and colleagues (2008) reported that impoverished inhibitory control among TTM patients predicted disorder severity and evolvement.

Second, the improvement of these two mindfulness facets suggests that the training modified the psychological processes of interest as intended. Moreover, it should be noted that FFMQ items refer to daily life situations rather than elements of the training sessions. Hence, our findings suggest that the changes induced by mindfulness training generalized to different types of situations. Our observation of an improvement in Describing and Nonreactivity to inner experience is also consistent with previous studies reporting that improvement on general psychopathological symptoms following mindfulness training were mediated by the increased capacities in these two facets only (Heeren et al., 2015). Results for the Nonreactivity facet are convergent with recent findings suggesting that improvement in top-down attention control, and more particularly inhibitory control, reduces psychological distress (Heeren, Coussement, & McNally, 2016; Houben, Wiers, & Jansen, 2011; Owens, Koster, Derakshan, 2013; Schweizer, Grahn, Hampshire, Mobbs, & Dalgleish, 2013). Results for the *Describing* facet are consistent with recent neuroscientific findings demonstrating that verbal labeling of affect modulates brain responses to emotional stimuli (Hariri, Bookheimer, & Mazziotta, 2000; Lieberman et al., 2007) and with evidence showing that a larger description of the details related to emotional experiences significantly reduces negative affect (Raes, Williams, & Hermans, 2009; Vrielynck, Philippot, & Rimé, 2010).

Third, more critically, our study showed that the mindfulness program had a beneficial effect transferred to hair-pulling severity. Even if the hair loss remission only occurred at six-month follow-up, a marginal trend to improvement was already evidenced at post-treatment. It is very likely that the eight-week period for treatment delivery has been too short to allow appropriate time to hair regeneration. Future studies should include more intermediate assessments of hair regeneration to better understand how this change occurs.

Importantly, the convergence of the clinical measures across the different times of assessment should be noted. This finding is critical because it suggests that the benefits of mindfulness training were not merely the mirror of error measurement or bias due to self-report assessment. At a process level, these findings suggest that, in the present case, improvement in mindfulness first occurred, and that this beneficial effect then transferred to hair-pulling. This bares critical implications vis-à-vis model of change and causality inference (e.g., Maurage, Heeren, & Pesenti, 2013)

At a clinical level, this study adds to a growing empirical literature suggesting that MBIs may be particularly suitable for treating individuals who suffer from TTM (Keuthen et al., 2010, 2011; Twohig & Woods, 2004; Woods et al., 2006b). Moreover, the present study is the first evidence that mindfulness training without HRT may lead to a significant improvement in TTM. This is particularly critical for future clinical studies with TTM. Indeed, although the extent of training is modest, totalizing no more than eight weekly sessions, significant clinical change occurred. Further, the six-month follow-up assessment revealed maintenance of these benefits. The present findings clearly suggest that randomized controlled trials are now needed to examine whether mindfulness training per se lead to superior symptoms reduction than the usual HRT treatment for TTM. Future studies might also examine the potential gains maintenance at one-year follow-up. They might also explore the potential long-term impact of adding a number of boosting sessions after the end of the treatment.

The present study also has some limitations. First, one cannot exclude that the improvement may be attributed to spontaneous recovery. Second, because of clinical constraints, the two people who administered the mindfulness training were not blind to the hypothesis of the present study. We could therefore not completely protect against the Rosenthal effect (Rosenthal & Rosnow, 1997), that one’s beliefs and expectations may influence the phenomenon under investigation. Finally, one cannot exclude that some idiosyncratic characteristics of DC (e.g., absence of comorbidity, motivation, educational level) might explain the present findings. Consequently, replications among other clients, including patients with comorbid disorders, are clearly needed to confirm the generalizability of the present findings.

In conclusion, the present findings have shown the efficacy of mindfulness training for TTM over a six-month follow-up period. Although it remains particularly difficult to infer generalization from one patient, the present data are the first to suggest that mindfulness training *per se* might be a suitable clinical intervention for TTM.

## Competing Interests

The authors declare that they have no competing interests.

## Acknowledgments

This research was supported by a Grant (Grant # FC 78142) from the Belgian National Science Foundation “F.R.S. –FNRS.” and by the Belgian Foundation for Vocation (“Vocatio”), both awarded to Dr. Alexandre Heeren. These foundations have no role in the study design, collection, analysis or interpretation of the data, writing the manuscript, or the decision to submit the paper for publication.

## Footnotes

It should be noted that he presence of two therapists is a common procedure of the clinical center where the present intervention took place (i.e., the Psychology Department Consulting Center of the Université catholique de Louvain, Belgium), rather than a specific strategy or concern vis-à-vis the present case study.
